# Emotional Eating and Uncontrolled Eating as Risk Predictors for Disordered Eating Attitudes in Candidates for Bariatric Surgery Treated at a Public Hospital in the Amazon

**DOI:** 10.3390/nu16111600

**Published:** 2024-05-24

**Authors:** Jeane Lorena Dias Kikuchi, Manuela Maria de Lima Carvalhal, Millena Borges Inete, Yasmym Dannielle do Espírito Santo Souza, Tainá Martins Moraes, Rafaela Lorena Viana Costa, Rafaelle Dias Gabbay, Carla Cristina Paiva Paracampo, Daniela Lopes Gomes

**Affiliations:** 1 Graduate Program in Neuroscience and Behavior, Behavior Theory and Research Center, Federal University of Pará, Belém 66075-110, Brazil; nutri.jeanedias@yahoo.com (J.L.D.K.); millenainete@gmail.com (M.B.I.); dannielleyasmym@gmail.com (Y.D.d.E.S.S.); tainamartmo@gmail.com (T.M.M.); rafaelaviananutri@gmail.com (R.L.V.C.); rafaellediasgabbay@gmail.com (R.D.G.); cparacampo@gmail.com (C.C.P.P.); 2 Social Service of Commerce, Belém 66010-010, Brazil; manuela.carvalhall@gmail.com

**Keywords:** obesity, bariatric surgery, eating behavior, eating disorders

## Abstract

This study analyzes the eating behavior and factors associated with the presence of disordered eating attitudes in patients undergoing bariatric surgery. It is a cross-sectional, descriptive, and analytical study conducted at a hospital in the Amazon region of Brazil. The Disordered Eating Attitude Scale reduced version (DEAS-s) was used to assess the risk of eating disorders and the Three-Factor Eating Questionnaire (TFEQ-R21) was used to characterize eating behavior. A total of 205 patients participated, with a mean age of 37.5 ± 8.6 years. The majority of participants were female (93.7%; *p* < 0.001), and the mean BMI was 45.3 ± 6.7 kg/m^2^. It was found that cognitive restraint had the highest mean (52.6 ± 19.9; *p* < 0.001). As for the DEAS-s, the question with the highest mean response was “spending one or more days without eating or consuming only liquids to lose weight” (2.80 ± 1.99). Female participants had a higher score for emotional eating (*p* = 0.016). Disordered eating attitudes showed a correlation with emotional eating and uncontrolled eating. These results suggest that candidates for bariatric surgery may have susceptibility to eating disorders. The importance of a multidisciplinary team conducting monitoring during the preoperative period is highlighted.

## 1. Introduction

Bariatric surgery has taken a prominent role in the treatment of severe obesity due to its sustained outcomes in weight loss and associated comorbidities [1]. However, adapting to new eating habits becomes a challenge during treatment, as the assessment of eating behavior patterns has often been neglected in traditional programs that focus solely on weight reduction without considering the psychological aspects involving food [2].

Some studies have focused on evaluating eating behavior in the preoperative period of bariatric surgery. Brode et al. [3] conducted a literature review aiming to assess problematic behaviors pre- and post-surgery, and to assess common eating disorders among these patients. They concluded that bariatric surgery candidates exhibited high rates of dysfunctional behaviors and/or eating disorders. 

Pekin et al. [4] investigated the rates of eating disorders in bariatric surgery candidates with type 2 diabetes, using instruments such as the Dutch Eating Behavior Questionnaire, a questionnaire that is very similar to the TFEQ-21, which assesses emotional eating, restrictive eating, and external eating, in addition to the Binge Eating Scale. In their results, they found that participants showed high scores of emotional eating and binge eating disorder, indicating that this population seems to be vulnerable to developing disordered eating attitudes.

Jesus et al. [5] determined, through the application of the Three-Factor Eating Questionnaire (TFEQ-R21), that emotional eating (eating in response to an emotional state) and food disinhibition (the presence of episodes of excessive food consumption in the presence or absence of hunger) were more prevalent in preoperative patients than in those who had already been operated on.

In a study by Cambiali et al. [6], which evaluated eating behavior in bariatric surgery candidates, the researchers found that emotional eating and food dependency were significantly correlated with the presence of disordered eating attitudes and anxiety in these individuals.

In an integrative review on eating disorders and disordered eating behaviors related to bariatric surgery, Novelle et al. [7] concluded that dysfunctional eating behaviors are very common in bariatric surgery candidates and may persist or even worsen after surgical treatment.

Marek et al. [8] tested the association between psychosocial functioning (stress, anxiety, impulsivity, etc.), using the Minnesota Multiphasic Personality Inventory (MMPI-3), and weight regain and other behaviors. It was observed that higher scores on the MMPI-3 were associated with disordered eating behaviors and weight regain 6 years after surgery.

Thus, it is understood that this population demands attention in the analysis of eating behavior, in addition to monitoring food consumption and habits to promote negative energy balance and weight loss [9].

With the aim of evaluating eating attitudes, Alvarenga et al. [10] developed the Disordered Eating Attitude Scale (DEAS), which assesses attitudes towards food encompassing beliefs, thoughts, and feelings related to food, where higher scores indicate more dysfunctional eating attitudes.

Simões et al. [11] conducted a retrospective study on 281 bariatric surgery candidates and observed, from electronic records, that 26.7% of participants were diagnosed by specialists as having an eating disorder. These data demonstrate the importance of identifying signs of eating disorders in the preoperative period, as they can interfere with eating behavior and, consequently, surgical outcomes.

In this context, it is noteworthy that no research was found that applied the DEAS-s to bariatric surgery candidates, and few researchers used it in people with obesity. Therefore, due to the vulnerability of this population to disordered eating behaviors and the increasing number of bariatric surgery candidates and scarcity of investigations using this tool, it is important to assess whether bariatric surgery candidates exhibit disordered eating behaviors that may predict the development of eating disorders.

Thus, the present study aimed to analyze the characteristics of eating behavior and factors associated with susceptibility to the presence of disordered eating attitudes in patients undergoing bariatric surgery followed at a public hospital specializing in severe obesity care in the Amazon, Brazil.

## 2. Materials and Methods

### 2.1. Study Type and Ethical Aspects

Our study was a cross-sectional, descriptive, and analytical study conducted at a public hospital in Belém, in the Amazon region of Brazil. The study was approved by the Ethics Committee on Human Research of the Nucleus of Tropical Medicine of the Federal University of Pará (opinion n° 5.180.990), and according to the Helsinki Declaration. All participants signed an Informed Consent Form (ICF).

### 2.2. Participants

Participant recruitment took place at the Endocrinology Outpatient Clinic of the Jean Bitar Hospital (HJB). A total of 205 participants were evaluated, corresponding to 100% of the estimated sample. Sample calculation was based on the number of surgeries performed at the hospital, with a margin of error of 5% and confidence level of 95%. The study included individuals of both sexes aged 18 to 64 years, who were candidates for bariatric surgery, followed at the endocrinology outpatient clinic of HJB, who agreed to participate in the research by signing the ICF. Literate individuals who did not have impaired understanding of the research and writing were included.

Individuals under the age of 18 and over the age of 64 were excluded, as were those with illnesses that could potentially affect body weight or eating behavior. Additionally, those diagnosed with an eating disorder and those who did not accurately complete all questionnaires were also excluded.

### 2.3. Data Collection

Data collection took place between February and September 2022. Participants were approached in the waiting room of the clinic and the research procedure was explained to them. Subsequently, a sociodemographic questionnaire was administered, and measurements of weight and height were taken. Later, the DEAS-s instruments were used to assess the risk of eating disorders and the TFEQ-21 to characterize eating behavior patterns, with a duration of 20 min. Anthropometric evaluation was conducted using a platform-type scale to measure weight and a stadiometer to measure height [12,13].

### 2.4. Instruments

The DEAS-s, in its reduced version, was developed by Alvarenga et al. [14]. Its aim is to observe and assess beliefs, feelings, thoughts, and behaviors associated with eating, as well as the individual’s relationship with food. The abbreviated version of the questionnaire consists of 17 items, a reduction from the original 25-item version. In addition to reducing the number of questions, the short version provides a more precise measure and adds a description of eating disorder attitudes at each level of the continuum. The items in this questionnaire are organized for dichotomous response and Likert scale, which consists of a scoring system.

The TFEQ-21 reduced version was translated and validated for the Brazilian population by Natacci et al. [15]. This instrument evaluates three behavioral aspects of eating: emotional eating, cognitive restraint, and uncontrolled eating. Emotional eating comprises 6 questions and refers to eating in response to an emotional state, or when a person’s behavior changes due to changes in mood or challenging circumstances. Cognitive restraint is characterized by dietary restriction with the intention of losing weight or fear of gaining weight; it also consists of 6 questions addressing obligations, prohibitions, and dietary restrictions. Uncontrolled eating investigates the propensity to lose control over one’s eating and occurs when a person engages in excessive food consumption in the presence or absence of hunger or when exposed to an external stimulus. This part of the questionnaire comprises 9 questions.

### 2.5. Data Analysis

The Statistical Package for the Social Sciences software, version 21.0, was used to perform the statistical analysis. Descriptive results were presented in measures of central tendency and dispersion. The Kolmogorov–Smirnov normality test was applied. The Mann–Whitney test was used to compare male and female groups. Bivariate correlations were conducted using the Spearman correlation test, and variables showing statistically significant correlations were included in the multiple linear regression model. The statistical significance level considered was *p* < 0.05.

## 3. Results

A total of 205 patients participated in the study, with a mean age of 37.5 ± 8.6 years, with the majority being female (93.7%; *p* < 0.001). The mean BMI was 45.3 ± 6.7 kg/m^2^, with most participants having grade III obesity (78%; *p* = 0.063). The characterization of the socioeconomic and demographic profile has already been described by Kikuchi et al. [16]; their work is the first publication of a larger project on eating behavior of bariatric surgery candidates.

Regarding eating behavior assessed through the TFEQ-21, it was found that the cognitive restraint domain had the highest mean (52.6 ± 19.9; *p* < 0.001), followed by the emotional eating domain, with a mean score of 41.1 ± 30.3 (*p* < 0.001) (Table 1).

As for the DEAS-s, the category with the highest mean was “spending one or more days without eating or consuming only liquids to lose weight” (2.80 ± 1.99), and the category with the lowest mean was “counting calories for everything you eat” (Table 2).

In the bivariate correlation analysis, emotional eating showed a positive correlation with uncontrolled eating (r^2^ = 0.640; *p* < 0.001) and the score of disordered eating attitudes (r^2^ = 0.280; *p* < 0.001). Additionally, the cognitive restraint domain correlated with the emotional eating domains (r^2^ = −0.334; *p* < 0.001) and uncontrolled eating (r^2^ = −0.383; *p* < 0.001); the uncontrolled eating domain also correlated positively with the score of disordered eating attitudes (r^2^ = 0.349; *p* < 0.001), as shown in Table 3.

When comparing eating behavior between genders, it was observed that female participants had a higher score in the emotional eating domain (*p* = 0.016), with no statistically significant difference for the other parameters (Table 4).

In the multiple linear regression analysis between the score of disordered eating attitudes and the emotional eating domain, it was observed that emotional eating (β = 0.336; CI = 0.005; 0.011; *p* < 0.001) emerged as a predictor for disordered eating attitudes, independent of gender (Table 5).

Similarly, it was observed that uncontrolled eating (β = 0.395; CI = 0.009; 0.018; *p* < 0.001) also emerged as a predictor for disordered eating attitudes, independent of gender (Table 6).

## 4. Discussion

The present study evaluated the characteristics of eating behavior and factors associated with susceptibility to disordered eating attitudes in patients undergoing pre-bariatric surgery follow-up at a public hospital specializing in severe obesity care in the Amazon region of Brazil.

In this study, cognitive restraint was the most frequent domain among participants, followed by emotional eating and uncontrolled eating. This is consistent with the findings of Wong et al. [16], who also applied the TFEQ-21 in bariatric surgery candidates.

Cognitive restraint was also the most frequent eating behavior pattern in patients studied by Conceição et al. [17] before bariatric surgery, followed by emotional eating and uncontrolled eating. This possibly reflects the level of interest of this group in reducing food intake, which is likely encouraged by the imminent surgical intervention. The same study also assessed disordered eating behaviors using the Eating Disorder Examination Questionnaire (EDE-Q), and the scales with the highest scores were weight concern and shape concern scales.

This contrasts with the study conducted by Aymes et al. [18], which explored associations between TFEQ-21 eating behavior domains in individuals with and without obesity. It was observed that all three eating behavior domains were higher in participants with obesity, but emotional eating stood out the most. However, this population did not consist of bariatric surgery candidates.

Similarly, research conducted by Parker et al. [19], using the TFEQ-21 in bariatric surgery candidates, revealed that the majority of participants exhibited uncontrolled eating, followed by emotional eating. The findings of the above authors differ from our results, suggesting that emotional eating and uncontrolled eating are more strongly associated with obesity. It is hypothesized that the difference in results may derive from cultural variability, methodologies used, the form of assistance, and access to information that these populations may have received before the studies.

According to Bryant et al. [20], dietary restraint can also result in weight gain and obesity by promoting increased food cravings. Restraint may lead to a heightened hedonic response to food, and consequently, increased caloric intake. This is probably because, in an obesogenic environment, food pleasure may prevail over the goal of weight control, leading to unhealthy eating patterns.

Regarding the DEAS-s, the category with the highest mean was “spending one or more days eating or consuming only liquids to lose weight”. According to Aparicio-Martinez [21], these attitudes may be associated with excessive thoughts about food and one’s diet, dysfunctional beliefs, and emotions related to food, which are strongly linked to the development of eating disorders.

Applying the DEAS-s questionnaire in overweight women, Alvarenga et al. [14] observed that women between 20 and 30 years old and with a higher BMI demonstrated a greater presence of disordered eating attitudes. However, no studies were found that applied the DEAS-s questionnaire in bariatric surgery candidates.

Correlation analyses indicated that as emotional eating increases, so does uncontrolled eating and the score of disordered eating attitudes. Moreover, in multiple linear regression analysis, it was observed that the score of disordered eating attitudes was correlated with emotional eating and uncontrolled eating, regardless of gender. Emotional eating was also correlated with the domain of uncontrolled eating, corroborating the research by Natacci et al. [15] and Biagio et al. [22], who identified positive correlations between these domains.

Another study involving individuals seeking bariatric surgery evaluated eating behavior, comorbid psychiatric conditions, and impulsivity, and found significant correlations between emotional eating and depression, anxiety, and impulsivity. There was also a correlation between external eating (triggered by the sensory characteristics of food) and depression, anxiety, lack of perseverance, and sensation seeking [23].

Sarwer et al. [24] evaluated psychosocial functioning (symptoms of depression, anxiety, trauma, etc.), impulsivity, and eating behavior in patients seeking bariatric surgery and identified that psychiatric symptoms were associated with eating disorder symptoms and impulse control problems.

Bryant et al. [20] observed that in individuals with obesity, uncontrolled eating is strongly related to lower stress tolerance, exacerbating the response to overeating, and is closely linked to eating disorder symptomatology. According to Konttinen [25], individuals with high scores on scales assessing emotional eating may consume more high-energy-density foods after experiencing negative emotions as a way of coping with stress and other feelings.

Parker et al. [19] found that cognitive restraint was negatively correlated with uncontrolled eating and emotional eating, which was similar to the findings in this study. These results may be explained by the higher prevalence of women in our sample, as women, in general, commonly suffer from stigma surrounding their physical appearance and constant concern about their weight [26], and thus are more emotionally susceptible to developing disordered eating practices as a compensatory mechanism [27].

Ernst et al. [28] analyzed the eating behavior of obese patients and observed that women, compared to men, showed higher scores in the domain of cognitive restraint, followed by emotional eating and uncontrolled eating, respectively. Another study, which assessed the eating behavior of adolescents and young adults referred for obesity treatment, also found that women had higher scores than men in the domain of emotional eating [29].

The present study has some limitations, such as the collection of some information through self-reports, as well as the limitation of the cross-sectional design conducted in just one hospital, making it impossible to establish a cause-and-effect relationship. It is also important to note that the data presented here correspond to the preoperative period, a time in which patients are immersed in the guidance received at this stage. Furthermore, the imminence of surgery can cause changes in mood and eating behavior in this population. Additionally, no information was collected about psychological distress, which would be useful for analyzing the nature of negative emotions associated with eating behavior.

The study was conducted in the Amazon region of Brazil, at a public hospital, where healthcare is scarce. Furthermore, to access this high-complexity service, the patients needed to have a level of independence and the ability to access information. Therefore, caution is necessary when generalizing the results found in this study and it is suggested that studies with greater representation of the Brazilian population be carried out in the future.

However, this research may assist in clinical practice interventions, indicating a direction for future experimental studies. Furthermore, it is essential for future research to compare pre- and post-operative data, as well as intervention studies analyzing eating behavior before bariatric surgery, due to the scarcity of research related to this topic.

No other studies were found that incorporated the questionnaire used to assess disordered eating attitudes in this population, demonstrating the importance of this research. The responses to the DEAS-s provide some insights into the strategies that this population uses to pursue weight loss and the challenges encountered on this journey, such as negative emotions related to eating and limited knowledge about the nutritional composition of foods.

Given that eating is possibly associated with emotional conditions that this group is either unaware of or lacks the tools to cope with, cognitive restriction manifests through engagement in health-damaging behaviors to achieve quick and visible results. These behaviors include fasting for one or more days, skipping meals, and replacing meals with liquids. These responses to the DEAS-s found in our study can be used to guide the conduct of health professionals working in the field and help propose alternatives to these maladaptive behaviors.

## 5. Conclusions

A higher prevalence of cognitive restraint domain was observed, while women showed a higher score in the emotional eating domain. Disordered eating attitudes showed a positive correlation with the emotional eating domain and uncontrolled eating, regardless of gender. These results suggest that candidates for bariatric surgery may have susceptibility to eating behavior disorders. The importance of a multidisciplinary team in monitoring during the preoperative period is highlighted in order to prevent and treat possible dysfunctional behaviors.

## Figures and Tables

**Table 1:** Eating behavior characterization of candidates for bariatric surgery.

	n/Mean ± SD *	Interval/%	Value of *p* **
Dietary pattern			
Cognitive restriction	52.6 ± 19.9	5.56–100.0	<0.001 ***
Emotional eating	41.1 ± 30.3	0–100.0	<0.001 ***
Uncontrolled eating	37.9 ± 21.0	0–100.0	<0.001 ***

* Standard deviation. ** Qui-square. *** Friedman test.

**Table 2:** Description of the mean and standard deviation per subscale of the DEAS-s in candidates for bariatric surgery.

	Mean	SD *
Not eating or consuming only liquids	2.80	1.99
Skips meals	2.62	1.97
Relationship with food	2.52	1.95
Feels guilty	2.50	1.00
Worries about how much a food will make gain weight	2.42	1.10
Finds it hard to choose what to eat	2.38	1.03
Feels angry when feeling hungry	2.37	1.90
Overeats	2.36	1.11
Would like to not need to eat	2.33	1.89
Feels “dirty”	2.31	1.88
Thinks eating feels unnatural	2.11	1.80
Dreams of a pill that would replace food	2.01	1.75
Will quit eating a food if it has more calories	1.85	0.95
Uncontrolled eating	1.80	1.49
Overeats when alone	1.78	1.58
Binge eating	1.66	1.00
Counts calories	1.34	1.11

* Standard deviation.

**Table 3:** Bivariate correlation analysis between different patterns of eating behavior and presence of disordered eating attitudes in candidates for bariatric surgery.

	*r* ^2^	Value of *p* *
Emotional eating		
Disordered eating attitudes	0.280	<0.001
Uncontrolled eating	0.640	<0.001
Cognitive restriction		
Emotional eating	−0.334	<0.001
Uncontrolled eating	−0.383	<0.001
Uncontrolled eating		
Disordered eating attitudes	0.349	<0.001

* Spearman’s correlation test, statistical significance *p* < 0.005.

**Table 4:** Eating behavior and presence of disordered eating attitudes by sex in candidates for bariatric surgery.

	Female (*n* = 192)		Male (*n* = 13)		
Eating Behavior	Mean ± DP	Median (P25-P75)	Mean ± SD	Median (P25-P75)	Value of *p* *
Cognitive restriction	53.1 ± 20.1	55.5 (38.8–66.6)	45.7 ± 17.4	50.0 (22.2–77.7)	0.169
Emotional eating	42.3 ± 30.1	38.8 (16.6–66.6)	23.0 ± 28.0	16.6 (0.0–88.8)	0.016
Uncontrolled eating	38.3 ± 21.1	37.0 (22.2–51.8)	31.9 ± 18.4	25.9 (20.3–35.1)	0.187
Disordered eating attitudes	2.1 ± 0.7	2.1 (0.0–4.4)	1.9 ± 0.6	1.8 (1.4–2.5)	0.307

* Mann–Whitney test.

**Table 5:** Multiple linear regression between DEAS-s score and the factor of emotional eating in candidates for bariatric surgery.

DEAS-s Score	*B* *	CI 95% (Minimum; Maximum)	Value of *p* **
Model 1			
Emotional eating	0.339	0.005; 0.011	**<0.001**
Model 2			
Emotional eating	0.336	0.005; 0.011	<0.001
Gender	−0.021	−0.458; 0.333	0.757

* Regression coefficient. ** Linear regression. Dependent variable: DEAS-s; co-variables: emotional eating, uncontrolled eating, and gender. Bold values indicate statistical significance.

**Table 6:** Multiple linear regression between DEAS-s score and the factor of uncontrolled eating in candidates for bariatric surgery.

DEAS-s Score	*B* *	CI 95% (Minimum; Maximum)	Value of *p* **
Model 1			
Uncontrolled eating	0.398	0.009; 0.018	<0.001
Model 2			
Uncontrolled eating	0.395	0.009; 0.018	<0.001
Gender	−0.044	−0.512; 0.251	0.500

* Regression coefficient. ** Linear regression. Dependent variable: DEAS-s; co-variables: emotional eating, uncontrolled eating, and gender.

## Data Availability

Data are available upon request due to restrictions aimed at preserving the privacy of the participants. The data presented in this study are available upon request to the project’s research coordinator. The data are not publicly available due to ethical considerations regarding the preservation of participants’ identities.
